# Local Diagnostic Reference Levels for Adult Computed Tomography Urography Exams

**DOI:** 10.3390/diagnostics14060643

**Published:** 2024-03-19

**Authors:** Faruk Husremović, Orhan Muharemović, Edis Đedović, Alma Efendić, Jasmin Mušanović, Rifat Omerović, Hedim Osmanović, Mustafa Busuladžić

**Affiliations:** 1 Department of Urology, Cantonal Hospital Zenica, 72000 Zenica, Bosnia and Herzegovina; faruk.husremovic@gmail.com; 2 Department of Radiology, Cantonal Hospital Zenica, 72000 Zenica, Bosnia and Herzegovina; oki91ze@gmail.com (O.M.); alma_efendic@yahoo.com (A.E.); 3 Independent Researcher, London SM1 4BB, UK; edis.djedovic@yahoo.com; 4 Faculty of Medicine, University of Sarajevo, 71000 Sarajevo, Bosnia and Herzegovina; jasmin.musanovic@mf.unsa.ba; 5 Faculty of Natural Sciences and Mathematics, University of Tuzla, 75000 Tuzla, Bosnia and Herzegovina; rifat.omerovic@untz.ba (R.O.); hedim.osmanovic@untz.ba (H.O.)

**Keywords:** computed tomography, urography, dose descriptors, diagnostic reference levels

## Abstract

A Computed Tomography Urography (CTU) scan is a medical imaging test that examines the urinary tract, including the bladder, kidneys, and ureters. It helps diagnose various urinary tract diseases with precision. However, patients undergoing CTU imaging receive a relatively high dose of radiation, which can be a concern. In our research paper, we analyzed the Computed Tomography Dose Index (CTDI_vol_) and Dose-Length Product (DLP) for 203 adult patients who underwent CTU at one of the most important regional centers in Bosnia and Herzegovina that sees a large number of patients. Our study included the distribution of age and sex, the number of phases within one examination, and different clinical indications. We compared our findings with the results available in the scientific literature, particularly the recently published results from 20 European countries. Furthermore, we established the local diagnostic reference levels (LDRLs) that can help set the national diagnostic reference levels (NDRLs). We believe our research is a significant step towards optimizing the protocols used in different hospitals in our country.

## 1. Introduction

The recent advancements in Computed Tomography (CT), particularly in image reconstruction techniques, have introduced additional opportunities to enhance the benefit–risk ratio for patients undergoing this examination. A comprehensive benefit–risk analysis in medical diagnostic X-ray usage is a highly intricate task encompassing multiple facets [1]. The primary risk component arises from the adverse effects of ionizing radiation on a patient’s health, which correlates with the dose delivered to patients during an examination. The secondary risk component is associated with potential misdiagnosis, largely stemming from inadequate image quality, consequently leading to inappropriate therapy for patients. The central objective of the ALARA principle (As Low as Reasonably Achievable) is to strike a balance between image quality and the radiation dose received by patients during an examination. This principle emphasizes the need to optimize the benefit–risk ratio and enhance radiological protection for patients against ionizing radiation.

The initial step in achieving the optimal benefit-to-risk ratio involves investigating the dosimetric impact of CT examination protocols. Paying particular attention to doses is crucial, considering that CT contributes to 41% of the total population’s radiation dose [2]. Furthermore, the significance of this attention lies in the doses received by patients during CT examinations, which reach a level posing a genuine risk of radiation-induced cancer [3]. It is crucial to acknowledge that younger patients are more susceptible to the effects of radiation compared to older individuals. This heightened sensitivity is due to their longer lifespan, allowing radiation effects more time to manifest. Among all age groups, children are particularly sensitive as their bodies are in the developmental phase. Epidemiological studies have assessed radiation exposure from childhood CT scans and the associated cancer risks [4,5]. Consequently, maintaining a balance between the utility of CT scans and safeguarding the health of younger patients, especially children, holds paramount importance.

The *European Commission Directive* 2013/59/EURATOM [6] and other documents from international advisory groups [7,8,9] underscore the importance of establishing diagnostic referent levels (DRLs) for patients undergoing X-ray diagnostic and/or interventional procedures. The *European Commission Report 180* [10] contains DRL values for adult X-ray examinations in 36 European countries and pediatric X-ray examinations in 14 European countries. Establishing DRLs at local, regional, and national levels serves as a strong foundation for standardizing practices and enhancing the radiological protection of patients. These DRLs are derived from dosimetric values obtained across various X-ray modalities, anatomical regions examined, clinical indications, and patient age groups, gathered through surveys involving standard-size patient groups at local or regional levels. They provide a basis for setting national DRLs. Moreover, DRLs are indispensable tools in the process of optimizing doses for X-ray examinations.

This paper focuses on analyzing the dose descriptors associated with Computed Tomography Urography (CTU), a form of abdominal multiphase CT scan utilized for imaging the kidneys, ureters, and bladder [11]. Over the past two decades, CTU has emerged as a crucial imaging technique for evaluating the urinary tract, offering precise diagnostics for various pathologies like hematuria, urolithiasis, and bladder cancer. For more comprehensive information, readers can refer to the review article and its references [12]. However, CTU exposes patients to higher levels of radiation. The scientific community has diligently worked to optimize different protocols within CTU imaging techniques [13,14]. Unfortunately, there is no consensus on a standard or suitable protocol for clinical indications [15]. CTU commonly involves scanning in 2–6 different phases, both with and without intravenous contrast administration. Presently, the most frequently used and suggested protocols include a two-phase split bolus CTU, comprising a non-contrast phase and a combined nephrographic and excretory phase, as well as a three-phase protocol based on a single bolus, encompassing non-contrast, nephrographic, and excretory phases [16]. The split bolus protocol has demonstrated a reduction in delivered patient doses. Obtaining a clear image can be challenging due to delayed excretion or suboptimal display of the channel system. At times, increasing doses becomes necessary for improved image quality. Balancing this, however, is the significance of limiting ionizing radiation exposure to patients while ensuring satisfactory CTU image quality. A higher-quality image facilitates early detection and more reliable diagnoses [15].

A recent survey, conducted across various European countries by CTU, has revealed the use of diverse protocols and phases during CTU examinations [17]. This variability stems from differences in countries, regions/hospitals within countries, and the types of scanners utilized for CTU imaging. However, a lack of collaboration among medical physicists, radiologists, and technologists in certain sites can result in an increased number of phases conducted during a single examination, consequently elevating the patient’s radiation exposure. Notably, the survey encompassed only one regional center in Bosnia and Herzegovina, emphasizing the necessity for further analysis of fundamental dose descriptors within CTU examinations across other regional centers in the country.

This study aims to assess the local clinical practices at the Clinical Hospital Zenica concerning CTU examinations.

This effort is in accordance with the goal of the EUCLID study [18]. It intends to establish local diagnostic reference levels (LDRLs) and compare them with broader European Diagnostic Reference Levels (DRLs). Additionally, this research aims to provide insights into the gender and age demographics of patients and the clinical indications guiding referrals for CTU examinations. Utilizing the collected data and established LDRLs, we plan to develop assumptions for future dosimetric optimization of CTU imaging protocols. Moreover, the establishment of LDRLs holds the potential to drive the establishment of national Diagnostic Reference Levels (DRLs). Our primary motivation is to establish local diagnostic reference levels for CTU examinations at our hospital, aiming to inspire other regional centers across the country to adopt a similar approach, ultimately leading to the establishment of national diagnostic reference levels. Additionally, leveraging our findings, we intend to optimize our current protocols in line with the ALARA principle.

## 2. Materials and Methods

Data for CTU was sourced from Cantonal Hospital Zenica, one of Bosnia and Herzegovina’s prominent regional centers. The study encompassed 203 patients, comprising 91 females and 112 males. These patients were admitted to the regional hospital between mid-2019 and August 2023. CTU examinations were conducted using the SOMATOM Definition AS CT Scanner.

Patients underwent scans following a clinically adjusted CTU protocol known as the three-phase single-bolus protocol. Additional phases were occasionally included, particularly for older patients. The standard three-phase single-bolus protocol typically includes native, nephrographic, and excretory phases as part of the CTU examinations performed at our hospital [16]. The conventional CTU technique involves acquiring non-contrast images, administering the full contrast bolus, and then obtaining images in the nephrographic phase (80 to 120 s) and delayed excretory phase (5 to 15 min). Optionally, in certain cases, additional image acquisition in the corticomedullary phase (30 to 40 s) and/or late arterial phase may be conducted. At our institution, the excretory phase may be repeated in cases of complications or when obtaining essential diagnostic information becomes challenging. For this investigation and the corresponding statistical analysis, protocols consisting of four, five, and six phases were considered. Data were manually collected by urologists and radiologists and subsequently verified by all authors. Exclusions were made for pediatric cases and examinations with diagnostic uncertainty. Ethics approval was obtained from the institution’s ethics committee (Ethics Code: 00-03-35-1151-14/23). Given the retrospective nature of this study, patient consent corresponding to the Institutional Review Board of our hospital was not required.

CTU scans were conducted for 5 clinical indications: cystoscopically verified bladder tumor (18 patients), hydronephrosis of unclear etiology (51 patients), urolithiasis (30 patients), hematuria (15 patients), and ultrasound-verified tumoral changes in the kidney (89 patients). This classification aligns with domestic and international guidelines.

The primary focus of this study is the collection and analysis of CT dosimetric parameters, namely, the volumetric CT dose index (CTDI_vol_, measured in mGy) and the dose length product (DLP, measured in mGy·cm). The CTDI_vol_ refers to the CTDI32cm value and it is obtained as the weighted CTDI, CTDIw, normalized by the pitch value for helical scans [19]. It represents an estimate of the average dose within a scanned section in a standard CT 32 cm diameter circular PMMA phantom. It serves as a valuable standardized metric for comparing scanner outputs and optimizing protocols. The DLP value is defined as the product of CTDI_vol_ and scan length. The collected data about patient examinations include age, sex, clinical indication, date of examination, and the number of phases. The collected CT acquisition parameters include mAs (tube current-exposure time product), kV (tube voltage), Pitch Factor, Nominal Single Collimation Thickness, Nominal Total Collimation Width, Exposure Time per Rotation, and scan length. The automatic tube-current modulation in the angular and longitudinal directions was used. The acquisition parameters are listed in Table 1.

Although some parameters were changed during some phases within the same examination by CT technologists, such as the pitch factor, complete procedures are assumed to be part of one standardized protocol. We analyzed the dose descriptors in detail and found that there was no significant difference between the different protocols.

The analysis involved descriptive statistics such as arithmetic mean, median, interquartile range (IQR = 25th–75th percentile), and range values for CTDI_vol_ and DLP per phases and DLP_tot_. DLP_tot_ represents the cumulative sum of individual DLP values from each phase within a single examination. The values of CTDI_vol_, DLP, and DLP_tot_ were individually analyzed for examinations conducted with 3, 4, 5, and 6 phases, as well as their combinations (3 + 4, 3 + 4 + 5, and 3 + 4 + 5 + 6). Variations in radiation dose parameters were examined using box-and-whisker plots. The central line in the box represented the median value, while the edges of the box depicted the 25th–75th percentiles. The whiskers showcased the minimum and maximum values. The normality of the data was assessed using the Kolmogoro–Smirnov goodness-of-fit test. Additionally, the Kruskal–Wallis test was utilized to compare CTDI_vol_, DLP, scan length, and DLP_tot_ across different phases of the protocol. All statistical analyses were conducted using the R programming language, and statistical significance was indicated by *p* < 0.05. Table 2 provides a summary of CTU scan phases and obtained results for dose descriptors (CTDI_vol_, DLP, scan length and DLP_tot_).

## 3. Results

**Figure 1:**
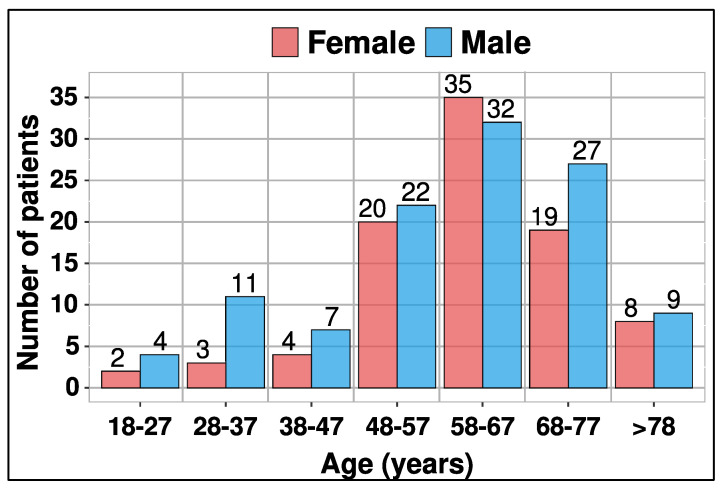
Age and sex distribution of patients who had CT urography from 2018 to 2023 in Cantonal Hospital Zenica. Total number of patients is 203.

A total of 203 patients participated in this retrospective study, comprising 91 females and 112 males. The difference in numbers between male and female patients was not statistically significant (*p* = 0.140, Mann–Whitney test). Patient ages ranged from 20 to 87 years and were categorized into 7 age groups, as depicted in Figure 1.

**Figure 2:**
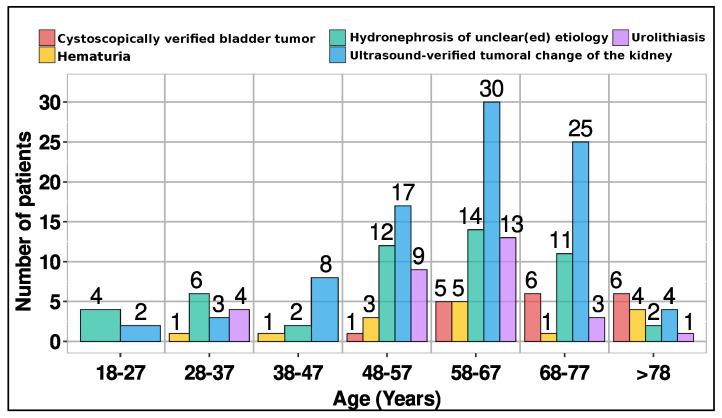
Distribution of clinical indications per age groups.

The mean age was 60.7 years (SD = 13.9), with a median age of 62 years. The 3- and 4-phase protocols were employed across a wider age range, spanning 20–87 and 21–85 years, respectively. These standardized protocols are widely utilized for various clinical indications (Figure 2).

**Figure 3:**
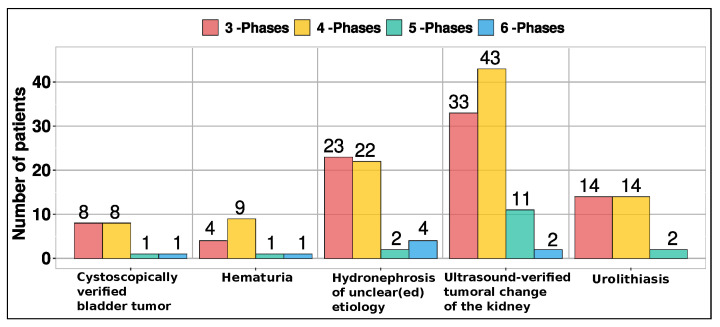
Phase distribution for various clinical indications.

The 5- and 6-phase protocols were administered to older patients, aged 42–82 and 60–81, respectively. Figure 3 presents the phase distribution for various clinical indications observed in CT urography.

Table 2 consolidates CTDI_vol_, DLP, scan length, and DLP_tot_ values per examination, offering insights into the range, mean (SD), median, as well as first and third quartile values for clarity.

**Figure 4:**
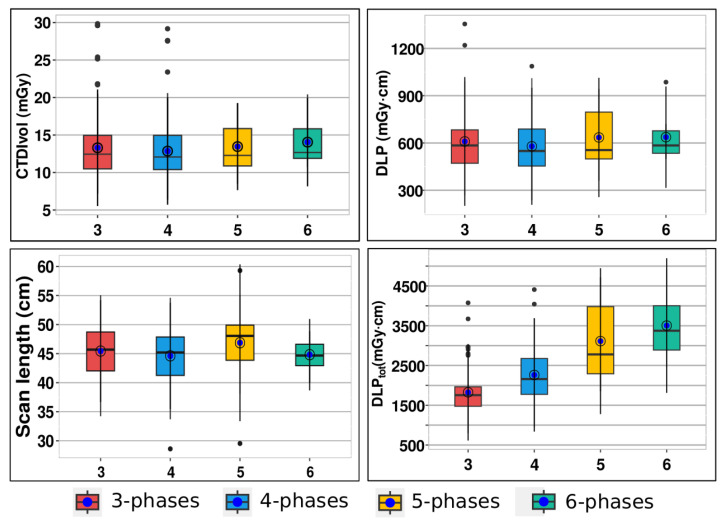
Box-and-whiskers plot for CTDI_vol_, DLP, scan length and DLP_tot_ per phases. For data set, whiskers present the full range of variations (minimum-maximum), box present 25th–75th percentiles, horizontal lines in box present median values of all scanners doses, and blue point present mean values (see Table 2).

The median number of scan phases for CTU stood at 4 (IQR 3–4 phases). Notably, CTU examinations were conducted using 3-phase (40.4%, 82/203), 4-phase (47.3%, 96/203), 5-phase (8.4%, 17/203), and 6-phase protocols (3.9%, 8/203). The median values for CTDI_vol_, DLP, scan length, and DLP_tot_ across the 3-phase, 4-phase, 5-phase, and 6-phase protocols are as follows: (12.44/12.07/12.28/12.67) in mGy, (584.5/549.3/555.0/584.5) in mGy·cm, (45.68/45.19/48.05/44.68) in cm, and (1755/2158/2779/3374) in mGy·cm, respectively. In Figure 4, we present box-and-whisker plots depicting CTDI_vol_, DLP, scan length, and DLP_tot_ per phase.

**Figure 5:**
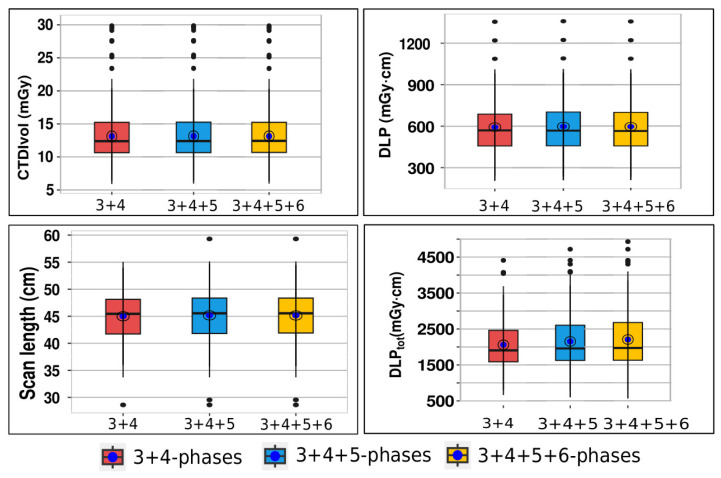
Box-and-whiskers plot for CTDI_vol_, DLP, scan length and DLP_tot_ across combined phases (3 + 4; 3 + 4 + 5; 3 + 4 + 5 + 6). For data set, whiskers present the full range of variations (minimum-maximum), box present 25th–75th percentiles, horizontal lines in box present median values of all scanners doses, and blue point present mean values (see Table 2).

Additionally, Figure 5 showcases CTDI_vol_, DLP, scan length, and DLP_tot_ across combined phases (3 + 4; 3 + 4 + 5; 3 + 4 + 5 + 6).

**Figure 6:**
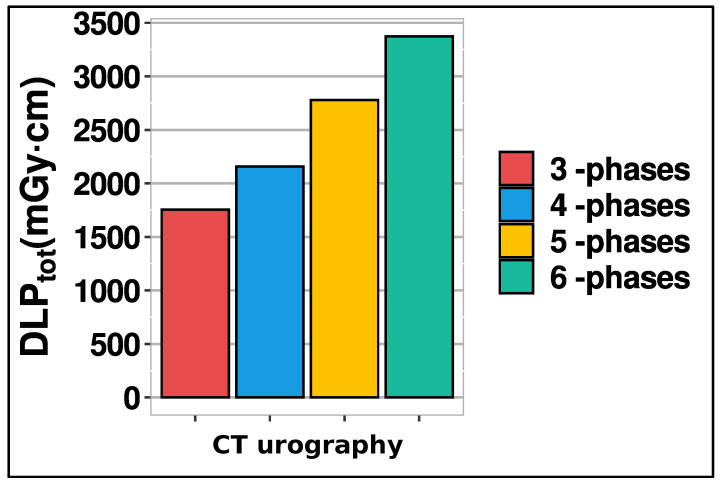
Bar diagram illustrates total DLP associated with different scan phases (see Table 2).

These calculations encompassed all doses from individual CT examinations, detailed in the previous section. From Figure 4, it is evident that there was no significant difference in CTDI_vol_, DLP scan length and DLP_tot_ values per phases between different protocols. Figure 6 displays the total DLP for 3, 4, 5, and 6 phases, revealing the 6-phase protocol’s DLP as 1.92 times higher than that of the 3-phase protocol.

In analyzing the distributions, mean values surpassing corresponding medians suggest skewed distributions for CTDI_vol_, DLP, and DLP_tot_. Conversely, the scan length distribution’s median and mean are closely aligned, indicating a relatively symmetric distribution. While the median of phase protocols for CTDI_vol_, DLP, and scan length showed no significant differences (*p* = 0.513, *p* = 0.503, *p* = 0.223, respectively), as expected, there was a significant disparity in the median of phase protocols for total DLP (*p* < 0.001).

## 4. Discussion

The research conducted at the Cantonal Hospital Zenica, followed by subsequent analysis, provided profound insights into CTU protocols, dose descriptors, patient demographics, and clinical indications. This enabled us to validate our clinical practices by establishing local diagnostic reference levels (LDRLs) for CTU examinations.

Data for 203 patients (91 females and 112 males) were gathered for this study. The study delves into the phases employed in CTU examinations, examining their correlation with clinical indications and patient age. The 3- and 4-phase protocols were employed across a wider age range, spanning 20–87 and 21–85 years, respectively. The primary reasons for undergoing this examination were ultrasound-verified tumoral changes in the kidney (43.8%, 89/203) and hydronephrosis of uncertain origin (25.1%, 51/203). Although ultrasound is commonly used for detecting kidney tumors, CT urography offers more precise information regarding tumor size, location, and features, aiding in staging and treatment planning. Hydronephrosis of unknown etiology can stem from diverse factors, including kidney stones, tumors, or congenital anomalies. Elderly individuals often present with multiple comorbidities, necessitating comprehensive examinations. Therefore, employing five- or six-phase protocols becomes crucial for providing accurate information vital in diagnosing and treating intricate medical conditions. In cases where the cause of hydronephrosis is unclear, CT urography is a preferred diagnostic tool due to its ability to offer detailed images of the urinary tract, aiding in identifying underlying issues. Other prevalent reasons for CT urography include urolithiasis (14.8%), cystoscopically verified bladder tumors (8.9%), and hematuria (7.4%). Urolithiasis, characterized by mineral and salt deposits in the urinary tract, is a global condition requiring imaging for diagnosis, follow-up, and management. CT urography provides crucial information for determining the stage of bladder tumors. Additionally, hematuria, indicating blood in the urine, could signify severe conditions such as bladder cancer, upper urinary tract urothelial cell carcinoma, renal cell cancer, or urinary tract stones. CT urography proves invaluable in diagnosing health issues related to hematuria.

The results indicate that there is no statistically significant variation in the numbers by gender. Due to men’s increased risk of urolithiasis and bladder cancer, an insignificant gender difference in favor of males was expected. The increased risk of urolithiasis in men is explained in part by the distinct chemical composition of urine and the presence of additional risk factors [20]. Additionally, women have a lower chance of bladder cancer due to prevention; while men have a two- or even three-fold higher risk of developing bladder cancer. One reason for this is that women have more frequent urological checks due to the higher incidence of urinary infections [21]. The number of patients undergoing CT examinations due to urinary tract issues (kidneys, ureters, and bladder) notably increased among individuals aged 48 years and older. The mean age was 60.7 years (SD = 13.9). According to the study [22], the peak age at which renal cancer is diagnosed is 50 years or older, and the mean age at which bladder cancer is diagnosed is approximately 64 years old, with most patients being over 50 years old. The main indications in our study were renal masses verified with ultrasound, hydronephrosis of unclear etiology, urolithiasis, bladder cancer verified cystoscopically and hematuria. These indications for clinical application of CT urography are align with the results of several previous studies [23,24].

The analysis revealed that mean values for CTDI_vol_, DLP, and scan length did not significantly differ between protocols. The existence of outliers may explain why mean values exceeded corresponding medians for CTDI_vol_ and DLP in the distributions. However, given the sample size, these outliers had minimal impact on the differences between mean and median values. As is common with LDRLs, our median values for CTDI_vol_ (12.44 mGy) and DLP (567 mGy·cm) across all protocols are selected as LDRLs for our hospital in CTU examinations. Comparatively, our study’s CTDI_vol_ median value of 12.44 mGy (with IQR 10.68–15.25) slightly exceeds the study [17], where the median CTDI_vol_ was 10 mGy (IQR 7–15) across 20 countries. While the study [17] didn’t list DLP per individual phases, our median DLP value (567 mGy·cm, IQR 460–701) aligns closely with the most common DLP (550 mGy·cm, range 450–650) for CT pelvis protocols in European countries [10]. Given the significant disparity in median DLP_tot_ values across protocols and the median of 4 phases for CTU examinations in our hospital, the LDRL value chosen for DLP_tot_ (2158 mGy·cm, IQR 1774–2676) corresponds to the protocol with 4 scanning phases. This value, higher than the study [17] median (1740 mGy·cm, IQR 869–2943), is because the study [17] accounted for CT scanners with iterative reconstruction capabilities, which tend to yield lower DLP_tot_ values. These findings underscore the necessity for standardized CTU protocols, balancing diagnostic accuracy with minimized radiation exposure.

The substantial difference in median DLP_tot_ values across protocols emphasizes the need for cautious optimization when employing multiple phases, notably the increased radiation dose exposure with a six-phase compared to a three-phase protocol. Due to the retrospective nature of the study, the patient’s habitus, in terms of BMI, could not be included in the analysis. We acknowledge this limitation as one of the weaknesses of our study. Nonetheless, we followed the recommendation outlined in ICRP Report 135 [19] suggesting that consideration of patient weight may not be necessary when a sufficiently large sample size (>100 patients) is obtained, as is the case in our study. In line with the primary objective of establishing local DRLs, we derived local DRL values from the analysis of collected data, adopting the median values of CTDI_vol_ and DLP as recommended practice [19], with minimal influence from outliers on the median distribution. Future initiatives should focus on optimizing CTU protocols, particularly for elderly patients who often require more phases, aiming to enhance diagnostic accuracy while reducing ionizing radiation exposure. In the optimization process, it is crucial to consider the patient’s habitus alongside assessing the image quality, ensuring alignment with the clinical task at hand. By incorporating these factors, CTU protocols should be optimized to accommodate patient habitus while effectively addressing clinical inquiries.

Additionally, given urolithiasis accounting for nearly 15% of CTU examinations, a specific CT protocol tailored for these examinations, as showed in study [17], is warranted. Collaboration among medical professionals across healthcare sites remains critical in standardizing CTU protocols, optimizing image quality, and reducing radiation doses, ultimately ensuring patient care and safety in diagnostic radiology.

We believe that established the local diagnostic reference levels (LDRLs) is a significant step towards optimizing the protocols used in different hospitals in our country. The establishment of national diagnostic reference levels (NDRLs) will benefit of this research.

## 5. Conclusions

The current study showed no significant difference between males and females who underwent CTU in our study. However, for both genders, the number of patients increased significantly above the age of 50, which is consistent with the results of other studies. According to similar investigations, almost 90% of the CTU exams consist of 3 or 4-phase protocols. The mean and median values of the dose descriptors were very close. The obtained median/mean values for CTDI_vol_, DLP, and DLP_tot_ slightly exceeded the values reported in a recently conducted investigation including results from 20 European countries. We also proposed a possible optimization of the protocol applied in our hospital. We did not analyze correlations of dose descriptors with BMI (body-mass index) due to a lack of information about the height and mass of patients. This is one of the most significant limitations of our investigation. Establishing a local diagnostic reference level (LDRL) is important step for standardizing radiation doses and guaranteeing patient safety. While our LDRLs provide valuable insights, their generalizability may be limited by single-center design. Multi-center studies are needed to potentially establish national DRLs. We intend to continue similar analyses in other hospitals and regional centers within the country to obtain the national diagnostic reference level (NDRL) as our final goal.

## Figures and Tables

**Table 1:** Scan protocol characteristics.

Tube Current-Exposure Time Product (mA·s) (Min–Max)	Ref. (mA·s)	Pitch Factor (Min–Max)	Tube Voltage (kV)	Time per Rotation (Second)	Nominal Single Collimation Thickness (mm)	Nominal Total Collimation Width (mm)	Exposure Modulation Type
87–407	210	0.6–1	120	0.5	1.2	1.92	XYZ_EC

**Table 2:** Summary of scan phases and associated radiation doses (CTDI_vol_, DLP, scan length and DLP_tot_) for the CT urography.

		3-Phases	4-Phases	5-Phases	6-Phases	3 + 4-Phases	3 + 4 + 5-Phases	3 + 4 + 5 + 6-Phases
Number of patients (total 203)		82	96	17	8	178	195	203
Percentage of exams		40.4	47.3	8.4	3.9	87.7	96.1	100
$\begin{array}{c} {\mathrm{CTDI}}_{\mathrm{vol}}\\ \left(\mathrm{mGy}\right) \end{array}$	Min	7.19	6.38	8.51	10.74	6.38	6.38	6.38
	Max	29.84	29.17	19.25	20.42	29.84	29.84	29.84
	Mean (SD)	13.31 (3.88)	12.85 (3.57)	13.46 (2.91)	14.04 (2.95)	13.03 (3.70)	13.08 (3.62)	13.14 (3.58)
	25th	10.49	10.39	10.88	11.88	10.48	10.56	10.68
	75th	14.95	15.00	15.86	15.83	14.95	15.16	15.25
	Median	12.44	12.07	12.28	12.67	12.29	12.28	12.44
$\begin{array}{c} \mathrm{DLP}\\ (\mathrm{mGy}\cdot \mathrm{cm}) \end{array}$	Min	267.0	237	361	498	237	237	237
	Max	1355	1150	948	998	1355	1355	1355
	Mean (SD)	608.26 (202.8)	578.8 (188.2)	622.5 (192.2)	637.9 (160.9)	585.1 (194.5)	596.8 (193.9)	598 (192.5)
	25th	471.5	453.9	499	534.9	457	457.5	460
	75th	683.3	688.6	796	677.3	687	701	701
	Median	584.5	549.3	555	584.5	569.5	567	567
$\begin{array}{c} \mathrm{Scan}\;\mathrm{Length}\\ \left(\mathrm{cm}\right) \end{array}$	Min	34.23	28.61	29.54	39.96	28.61	28.61	28.61
	Max	55.1	54.61	59.29	48.86	55.1	59.29	54.29
	Mean (SD)	45.4 (4.44)	44.6 (4.58)	48.05 (6.74)	44.82 (3.07)	44.9 (4.49)	45.5 (4.74)	45.15 (4.67)
	25th	42.03	41.25	43.86	42.93	41.73	41.82	41.91
	75th	48.72	47.86	49.90	46.61	48.13	48.36	48.36
	Median	45.68	45.19	48.05	44.68	45.43	45.55	45.54
$\begin{array}{c} {\mathrm{DLP}}_{\mathrm{tot}}\\ (\mathrm{mGy}\cdot \mathrm{cm}) \end{array}$	Min	799	948	1853	2450	799	799	799
	Max	4076	4409	4717	4928	4409	4717	4928
	Mean (SD)	1825 (606)	2261 (713)	3112 (918)	3504 (847)	2060 (698)	2152 (778)	2205 (821)
	25th	1474	1774	2292	2888	1588	1623	1629
	75th	1964	2676	3980	4002	2462	2602	2677
	Median	1755	2158	2779	3374	1905	1957	1970

## Data Availability

The data presented in this study are available upon request from the corresponding author.

## Abbreviations

The following abbreviations are used in this manuscript:

CTU	Computed Tomography Urography
CTDI_vol_	Computed Tomography Dose Index
DLP	Dose-Length Product
LDRL	Local Diagnostic Reference Levels
NDRL	National Diagnostic Reference Levels
ALARA	As Low as Reasonably Achievable

## References

[1] ICRP (1983). Cost-Benefit Analysis in the Optimization of Radiation Protection.

[2] United Nations Sources and Effects of Ionizing Radiation (2000). United Nations Scientific Committee on Effect of Atomic Radiation.

[3] Preston D.L., Shimizu Y., Pierce D.A., Suyama A., Mabuchi K. (2003). Studies of mortality of atomic bomb survivors. Report 13: Solid cancer and Noncancer disease mortality: 1950–1997. Radiat. Res..

[4] Mathews J.D., Forsythe A.V., Brady Z., Butler M.W., Goergen S.K., Byrnes G.B., Giles G.G., Wallace A.B., Anderson P.R., Guiver T.A. (2013). Cancer risk in 680,000 people exposed to computed tomography scans in childhood or adolescence: Data linkage study of 11 million Australians. BMJ.

[5] Berrington de Gonzalez A., Pasqual E., Veiga L. (2021). Epidemiological studies of CT scans and cancer risk: The state of the science. Br. J. Radiol..

[6] (2014). European Council Directive 2013/59/Euratom on basic safety standards for protection against the dangers arising from exposure to ionising radiation and repealing Directives 89/618/Euratom, 90/641/Euratom, 96/29/Euratom, 97/43/Euratom and 2003/122/Euratom. Off. J. Eu. L13.

[7] International Commission on Radiological Protection (1991). Recommendations of the International Commission on Radiological Protection. Ann. ICRP.

[8] Radiological protection and safety in medicine (1996). A report of the International Commission on Radiological Protection. Ann. ICRP.

[9] (2001). Diagnostic reference levels in medical imaging: Review and additional advice. Ann. ICRP.

[10] European Commission (EC) (2014). Radiation Protection No. 180–Diagnostic Reference Levels in Thirty-Six European Countries (Part 2/2). EC Website. https://www.eurosafeimaging.org/wp/wp-content/uploads/2015/05/Radiation-protection-180-part2.pdf.

[11] Molen A.J., Cowan N.C., Mueller-Lisse U.G., Nolte-Ernsting C.C.A., Takahashi S., Cohan R.H. (2008). CT urography: Definition, indications and techniques. A guideline for clinical practice. Eur. Radiol..

[12] Cellina M., Cè M., Rossini N., Cacioppa L.M., Ascenti V., Carrafiello G., Floridi C. (2023). Computed Tomography Urography: State of the Art and Beyond. Tomography.

[13] Kataria B., Nilsson Althén J., Smedby Ö., Persson A., Sökjer H., Sandborg M. (2019). Image quality and pathology assessment in CT Urography: When is the low-dose series sufficient?. BMC Med. Imaging.

[14] Rob S., Bryant T., Wilson I., Somani B.K. (2017). Ultra-low-dose, low-dose, and standard-dose CT of the kidney, ureters, and bladder: Is there a difference? Results from a systematic review of the literature. Clin. Radiol..

[15] Noorbakhsh A., Aganovic L., Vahdat N., Fazeli S., Chung R., Cassidy F. (2019). What a difference a delay makes! CT urogram: A pictorial essay. Abdom. Radiol..

[16] Morrison N., Bryden S., Costa A.F. (2021). Split vs. Single Bolus CT Urography: Comparison of Scan Time, Image Quality and Radiation Dose. Tomography.

[17] Gershan V., Homayounieh F., Singh R., Avramova-Cholakova S., Faj D., Georgiev E., Girjoaba O., Griciene B., Gruppetta E., Hadnadjev Šimonji D. (2020). CT protocols and radiation doses for hematuria and urinary stones: Comparing practices in 20 countries. Eur. J. Radiol..

[18] Damilakis J., Frija G., Jaschke W., Paulo G., Repussard J., Schegerer A., Tsapaki V., Clark J., Hierath M., European Commission, Directorate-General for Energy (2021). European Study on Clinical Diagnostic Reference Levels for X-ray Medical Imaging: EUCLID.

[19] Vañó E., Miller D.L., Martin C.J., Rehani M.M., Kang K.H., Rosenstein M., Ortiz-López P., Mattsson S., Padovani R., Rogers A. (2017). ICRP Publication 135: Diagnostic Reference Levels in Medical Imaging. Ann. ICRP.

[20] Ferraro P.M., Taylor E.N., Curhan G.C. (2023). Factors associated with sex differences in the risk of kidney stones. Nephrol. Dial. Transplant..

[21] Garg T., Pinheiro L.C., Atoria C.L., Donat S.M., Weissman J.S., Herr H.W., Elkin E.B. (2014). Gender disparities in hematuria evaluation and bladder cancer diagnosis: A population based analysis. J. Urol..

[22] Basiri A., Shakhssalim N., Jalaly N.Y., Miri H.H., Partovipour E., Panahi M.H. (2014). Difference in the incidences of the most prevalent urologic cancers from 2003 to 2009 in Iran. Asian Pac. J. Cancer Prev..

[23] Krishnan V., Chawla A., Sharbidre K.G., Peh W.C.G. (2018). Current Techniques and Clinical Applications of Computed Tomography Urography. Curr. Probl. Diagn. Radiol..

[24] Silverman S.G., Leyendecker J.R., Amis E.S. (2009). What is the current role of CT urography and MR urography in the evaluation of the urinary tract?. Radiology.

